# Numerical Evaluation on Analysis Methods of Trapping Site Density in Steels Based on Hydrogen Permeation Curve

**DOI:** 10.3390/ma13173712

**Published:** 2020-08-22

**Authors:** Bangshu Yang, Li Li, Lin Cheng

**Affiliations:** 1 The State Key Laboratory of Refractories and Metallurgy, Hubei Province Key Laboratory of Systems Science in Metallurgical Process, International Research Institute for Steel Technology, Collaborative Innovation Center for Advanced Steels, Wuhan University of Science and Technology, Wuhan 430081, China; yangbangshu2020@hotmail.com; 2 School of Mathematics and Computer Science, Hubei Key Laboratory of Digital Textile Equipment, Wuhan Textile University, Wuhan 430073, China; 3 Metals Valley and Band (Foshan) Metallic Composite Materials Co., Ltd., Foshan 528000, China

**Keywords:** hydrogen permeation, reversible trapping site, irreversible trapping site, numerical simulation

## Abstract

Hydrogen permeation techniques have been widely utilized to extract hydrogen effective diffusivity, as well as hydrogen trapping site characteristics in steels. Several methods have been proposed to examine reversible and irreversible trapping site characteristics. However, the inappropriate utilization of these simplified models, as well as incorrect value assignment to the key parameters, can result in several orders of magnitude difference in hydrogen trapping site density. Therefore, in order to evaluate these models and verify their application prerequisites, a serial of hydrogen permeation tests were numerically simulated and examined, separately considering reversible and irreversible hydrogen trapping sites. In the meantime, suggestions were given to conduct hydrogen permeation test more effectively, and evaluate hydrogen trapping site characteristics more precisely.

## 1. Introduction

In order to reduce resource consumption and improve the ecological environment, the successful development of ultra-high-strength steels, the development of modern zirconium-based, titanium-based and nickel-based alloys—that also face the problem of hydrogen embrittlement—and the implementation hydrogen energy economy to improve the global climate, make the study of hydrogen embrittlement mechanism in metallic materials and the design of anti-hydrogen embrittlement metallic materials (especially steels) a current research hotspot [1,2,3,4,5]. Hydrogen embrittlement susceptibility of structural steels increases with the yield strength of the steels, in general [6,7], for example, martensitic advanced high-strength steel [8,9]. Hydrogen effective diffusivity and hydrogen trapping site characteristics are the key to evaluate hydrogen embrittlement susceptibility of such advanced high-strength steels [10]. Since trapping sites can significantly decrease hydrogen effective diffusivity [6,11,12,13,14,15,16,17,18,19], permeation curves obtained by means of Devanathan–Stachurski cell [11,12] have been widely utilized to examine hydrogen effective diffusivity, as well as trapping site characteristics [6,11,12,13,15,16,17]. Generally, the hydrogen trapping site can be divided into reversible and irreversible types according to its strength level to bind with hydrogen. The former shows lower binding energy, and the latter exhibits higher binding energy. To separate the effects of reversible and irreversible trapping sites, at least two charging runs were suggested during the permeation test [20,21,22,23,24]. During the first permeation run, the irreversible trapping site was saturated, while during the second run, only the reversible trapping site was active. Meanwhile, partial build-up or decay transient methods were also suggested to eliminate the effects of specimen surface conditions on permeation curves [25,26]. Furthermore, in several advanced high-strength steels, it has been experimentally found that, with increasing hydrogen charging potential, the discrepancy between the successive normalized experimental permeation curves was continually narrowed [6,26]. This indicated that the effect of the trapping site on permeation curve was significantly decreased, and hydrogen diffusivity in matrix could then be obtained. However, researchers do prefer to utilize simple methods to examine hydrogen trapping site characteristics, based on one single complete permeation with one build-up or decay stage.

Besides fitting the experimental permeation curves, by means of the theoretical solution based on Fick’s diffusion law to obtain hydrogen effective diffusivity, the McNabb–Foster model [27] and Oriani’s local equilibrium theory [28] have also been proposed to integrate the trapping site’s effect on hydrogen diffusion behaviors in matrix, and establish the simplified relationship between trapping site characteristics and hydrogen effective diffusivity. With these theoretical foundations, two simplified formulas (Equations (1) and (2)) have been produced and widely utilized in the analysis of hydrogen permeation curves [22,26,29,30,31,32,33,34,35,36,37,38,39].

(1) $$D_{eff}=D_{L}{\left(1+\frac{N_{t}}{N_{L}}\exp\left(\frac{E_{B}}{RT}\right)\right)}^{{}_{-1}}$$

(2) $$N_{t}=\frac{C_{0}}{3}\left(\frac{D_{L}}{D_{eff}}-1\right)$$

where *D_eff_* is the hydrogen effective diffusivity examined in the experimental tests, *D_L_* is hydrogen diffusivity in matrix with trapping site effect; *N_t_* is trapping site density; *N_L_* is the density of interstitial site available for hydrogen in matrix; *E_B_* is the binding energy of hydrogen to trapping site and *C*_0_ is the hydrogen concentration at the charging side at hydrogen permeation test. Meanwhile, Equation (1) can be further transformed into

(3) $$N_{t}=N_{L}\left(\frac{D_{L}}{D_{eff}}-1\right)\exp\left(-\frac{E_{B}}{RT}\right)$$

Since the literature contains numerous disagreements about the values that should be assigned to parameters, such as *N_L_*, *D_L_* and *E_B_* [32], Equation (2) with fewer parameters became more popular to examine the so-called total trapping site density in the investigated steels. However, little attention has been paid to the application prerequisite of Equation (2) as well as the possibly inappropriate utilization of the decay part of normal permeation curves.

The magnitude of (reversible) hydrogen trapping site densities in steels, as well as pure irons, is reported to range from 10^18^/m^3^ to 10^32^/m^3^, which may be attributed to the intrinsic difference of the investigated materials and/or the methods difference for calculating the trapping site density [6,26]. It has been pointed out that the trapping site’s density could be examined in several orders of magnitude difference by assigning the wrong parameters or using inappropriate analysis formula [6,32]. For example, by analyzing permeation curves, the trapping site density in an X70 steel was examined as 1.3~3.1 × 10^18^/m^3^ [23]. However, in another X70 steel, the trapping site density was fitted as 2.81 × 10^27^/m^3^ [31]. Such a large difference could be accounted for in the use of incorrect simplified formulas [32]. In this present study, with the aim to bring new insights in the methods to evaluate hydrogen trapping site characteristics more precise, a series of numerical simulations were conducted. Firstly, the traditional methods to evaluate reversible and irreversible trapping density were introduced. Then, the accuracy of these simplified models was evaluated considering reversible and irreversible trapping sites, respectively. Finally, a new method was suggested to evaluate irreversible trapping density.

## 2. Methodology 

The McNabb–Foster model was utilized to simulate hydrogen diffusion behaviors incorporating hydrogen trapping site effects. To evaluate the application prerequisite of the traditional methods, the knowledge of the exact input parameters in the numerical simulation could eliminate the artificial error in fitting the experimental permeation curves. The McNabb–Foster model can be written as,

(4) $$\partial c_{L}/\partial t+N_{t}\partial \theta_{t}/\partial t=D\nabla^{2}c_{L}$$

(5) $$\partial \theta_{t}/\partial t=kc_{L}(1-\theta_{t})-p\theta_{t}$$

(6) $$k=k_{0}\exp(-\frac{E_{D}}{RT})$$

(7) $$p=p_{0}\exp(-\frac{E_{d}}{RT})=p_{0}\exp(-\frac{E_{B}+E_{D}}{RT})$$

(8) $$D_{L}=D_{0}\exp(-\frac{E_{D}}{RT})$$

where *c_L_* is hydrogen concentration in matrix; *N_t_* is trapping sites density; *θ_t_* is hydrogen occupancy in trapping site; *D_L_* is hydrogen diffusivity in matrix; *k* and *p* represent the probabilities of hydrogen jumping from lattice to trapping site and hydrogen detrapping from trapping site, respectively; *p*_0_ and *k*_0_ are the pre-exponential factors of *p* and *k*, respectively; *E_B_* is the binding energy of hydrogen to trapping site; *E_D_* is the activation energy hydrogen diffusion in matrix; *E_d_* is the activation energy of hydrogen detrapping from trapping site; and *D*_0_ is the pre-exponential factor of *D_L_*. 

The principle choice of the parameters follows our previous work [19]. Binding energies of 27 kJ/mol and 60 kJ/mol were chosen to represent reversible and irreversible trapping sites, respectively. For the lower binding energy, Oriani’s local equilibrium theory was assumed. The density of tetrahedral interstitial site for hydrogen accommodation in matrix was calculated as 5.08 × 10^29^/m^3^ assuming the lattice constant of the steel matrix equal to 2.85 × 10^−10^ m. Then, hydrogen trapping site density of 4.89 × 10^24^/m^3^ under saturation represents 1 part per million (ppm) hydrogen in weight percentage. Taking pipeline X70, for example, the hydrogen charging concentration underneath the hydrogen entry side during the electrochemical permeation was reported to vary in a large range of 0.603–134 mol/m^3^ [23], i.e., about (0.07–16.39) ppm in weight percent. Therefore, hydrogen charging concentration in present simulation was chosen around this range according to the simulation request.

In the present work, the permeation curves influenced by reversible or irreversible trapping site were first numerically simulated with the assigned input of trapping site and charging condition. Traditional methods were then utilized to examine trapping site characteristic parameters based on the simulated permeation curves. Finally, the accuracy of traditional methods was evaluated by comparing the examined and input characteristic parameters of the strapping site. 

## 3. Results and Discussion

### 3.1. Origin of the Simplified Formulas

To understand the origin of above-mentioned simplified formulas, in analysis of hydrogen effective diffusivity, one should start with the McNabb–Foster model. One can deduce from this model that the lag time (*t_T_*) of the specimen with a trapping site can be related to the lag time (*t_L_*), without considering trapping sites as [27,35]

(9) $$t_{T}=t_{L}\left\{1+\frac{3\alpha }{\beta }+\frac{6\alpha }{\beta^{2}}-\frac{6\alpha }{\beta^{3}}(1+\beta )\log(1+\beta )\right\}\;\mathrm{with}\;\alpha =N_{t}\frac{k}{p}\;\mathrm{and}\;\beta =C_{0}\frac{k}{p}$$

According to Equations (6) and (7), *α* and *β* can be rewritten as

(10) $$\alpha =\frac{N_{t}}{N_{L}}\exp(E_{B}/RT)\;\mathrm{and}\;\beta =\frac{C_{0}}{N_{L}}\exp(E_{B}/RT)$$

where *N_L_* represents the tetrahedral interstitial sites for hydrogen accommodation. To ease the application of this model to experimental data, two extreme cases, dilute occupancy of trapping site and near saturation of trapping site, were examined as

(11) $$\frac{t_{T}}{t_{L}}=1+\alpha =\frac{D_{eff}}{D_{L}}$$

(12) $$\frac{t_{T}}{t_{L}}=1+\frac{3\alpha }{\beta }=1+\frac{3N_{t}}{C_{0}}=\frac{D_{eff}}{D_{L}}$$

Substituting *α* into Equation (11), it can be further transformed into Equation (1). It is held assuming local equilibrium between hydrogen occupancy in matrix and hydrogen trapping site as well as a very limited hydrogen occupancy in trapping site. Substituting *α* and *β* into Equation (12), it can be transformed into Equation (2).

### 3.2. Irreversible Hydrogen Trapping Sites

**Figure 1:**
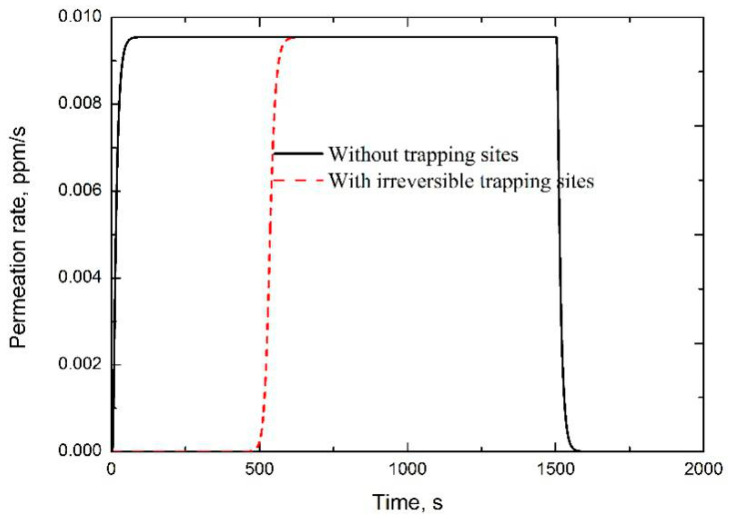
Influence of irreversible trapping sites on the permeation curve. An irreversible trapping site density of 4.89 × 10^25^ m^−3^ was assumed.

The irreversible hydrogen trapping site accommodates hydrogen until its saturation. Without changing the shape of the permeation curve, the irreversible trapping site simply delayed the whole permeation curve, as shown in Figure 1. Here, three permeation tests were simulated with different irreversible trapping densities or hydrogen charging concentrations. The first four rows of Table 1 show the parameters utilized in these simulations, as well as the correspondingly calculated values of 1 + *α* and 1 + 3*α*/*β*, based on these inputs. A trapping density of 0 cm^−3^ represents hydrogen diffusion simulation in pure matrix without considering the trapping effect. The lag time in pure matrix was examined as 17.8 s listed in the fifth row. The last row shows the ratio of lag time of the specimen with a trapping site to the one in pure matrix. It is clearly found that Equation (2) predicts the right ratio, comparing the third and sixth rows. However, Equation (1) gave totally wrong predictions, comparing the fourth and sixth rows. In another review work to evaluate hydrogen analysis methods utilized in steels by means of examining and fitting the experimental results [33], it was suggested that Equation (2) should be improved as

(13) $$N_{t}=\frac{C_{0}}{3}\left(\frac{D_{L}}{D_{eff}}-1\right)N_{A}$$

where *N_A_* is the Avogadro’s constant. *N_t_* is in the unit of cm^−3^, instead of mol/cm^−3^ for better understanding. With the same purpose, the former unit was utilized in this paper.

**Figure 2:**
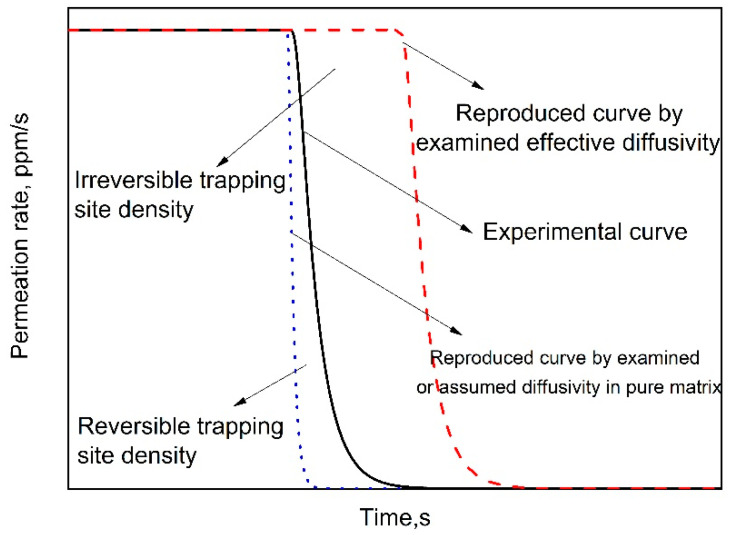
Proposed methods in literature to examine reversible and irreversible trapping side density at the decay process of hydrogen permeation curve.

The irreversible trapping site density was also suggested to be equal to the area difference between experimental permeation curve at the decay process and the one reproduced by examined effective diffusivity [29,36,40,41], as shown in Figure 2. That is to say, the irreversible trapping density should be equal to the area difference between the permeation curves without and with the trapping site at the build-up stage, as shown in Figure 1. In simulation of Figure 1, an irreversible trapping site density of 4.89 × 10^25^ was assumed. Therefore, 10.0 ppm hydrogen should be trapped, as the trapping site is saturated. However, the area enclosed by the two simulated permeation curves in Figure 1 only accounts for about 5.0 ppm hydrogen. Therefore, the above-mentioned method obviously underestimated trapping site density because this method potentially assumes that hydrogen enters into the specimen with the constant flux at the steady state. However, the flux at the steady state should be lower than any others at the entry side before achieving steady state. Therefore, this method underestimates hydrogen amount absorbed into the specimen, i.e., the amount of hydrogen trapped in the irreversible trapping site, as well as its density.

### 3.3. Reversible Hydrogen Trapping Sites

**Figure 3:**
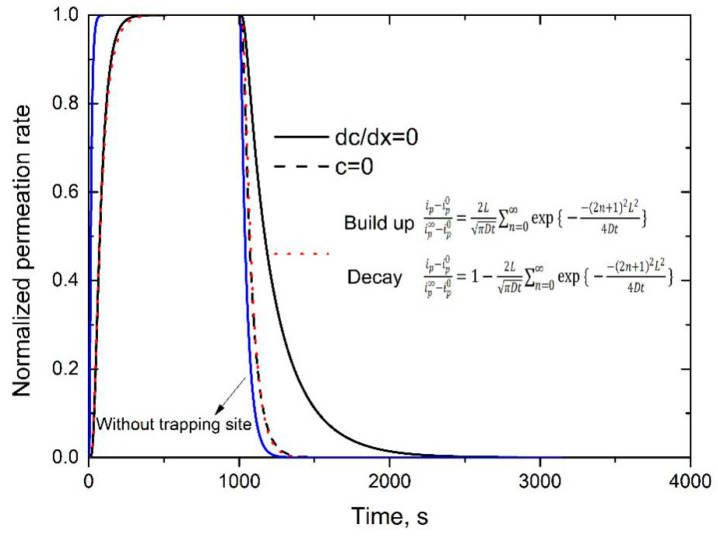
Simulated permeation curves with different boundary conditions at the initial entry side within the decay process. In this simulation, binding energy of 27 kJ/mol and trapping site density of 4.89 × 10^25^ m^−3^ were assumed. Hydrogen charging concentration at the entry side was assumed to be 1.0 ppm. After achieving steady state, hydrogen charging was turned off at 1000 s.

In Figure 3, the arrowed blue solid line is the hydrogen permeation curve in pure matrix, without considering the trapping site. The other dashed and solid lines show hydrogen permeation curves with the same trapping site density, but under two boundary conditions at the initial entry side during the decay process. The dotted line represents the reproduced permeation curve, by means of Fick’s diffusion law, under which permeation transients could be fitted by the following equations [18],

(14) $$\text{Build-up}\;\mathrm{transient}\;\frac{i_{p}-i_{p}^{0}}{i_{p}^{\infty }-i_{p}^{0}}=\frac{2L}{\sqrt{\pi Dt}}\sum_{n=0}^{\infty }\exp\left\{-\frac{{\left(2n+1\right)}^{2}L^{2}}{4Dt}\right\}$$

(15) $$\mathrm{Decay}\;\mathrm{transient}\;\frac{i_{p}-i_{p}^{0}}{i_{p}^{\infty }-i_{p}^{0}}=1-\frac{2L}{\sqrt{\pi Dt}}\sum_{n=0}^{\infty }\exp\left\{-\frac{{\left(2n+1\right)}^{2}L^{2}}{4Dt}\right\}$$

where *D* is hydrogen effective diffusivity examined at the build-up transient, and *L* is the thickness of the investigated specimen; *i_p_* is the measured current density at time (*t*) at specimen exit side; *i*^0^*_p_* is the initial steady-state current density (*t* = 0); *i*^∞^*_p_* is the new steady-state current density (*t*→∞), and *n* is the variable of summation series. In particular, for the complete decay process, *i*^∞^*_p_*= 0 and for the first hydrogen charging, *i*^0^*_p_*= 0.

Figure 3 demonstrates that permeation curves can be significantly delayed by hydrogen trapping sites. Meanwhile, even at the very early stage of the decay process, there is a significant discrepancy between the permeation curves without and with hydrogen trapping sites, which indicates that it could be not reliable to examine hydrogen diffusivity in pure matrix even at the very early stage of decay process. It is also found that Equations (14) and (15) fit very well with the simulated permeation curve. Here, it should be emphasized again that Equation (15) holds by assuming free boundary condition at the initial entry side within the decay process side (i.e., c = 0), which indicates that a large part of hydrogen could be released out at this side during the decay process [18]. Therefore, it is not accurate to examine the total reversible trapping site density by calculating the area enclosed by experimental permeation curve, and the one reproduced by hydrogen diffusivity in pure matrix (See Figure 2) [36,40]. Meanwhile, if assuming that hydrogen only diffuses out from the specimen exit side (i.e., dc/dx = 0 at the entry side), the permeation curve should be significantly delayed, as shown by the black solid one in Figure 3.

To evaluate the accuracy of Equations (1) and (2), in analyzing reversible trapping site density, four simulations with different trapping density and/or hydrogen charging concentration were conducted. The first four rows of Table 2 show the involved parameters, as well as the correspondingly calculated values of 1 + *α* and 1 + 3*α*/*β*, based on these inputs. The fifth row shows the calculated hydrogen occupancy in trapping site near the entry side in each simulation, based on Oriani’s local equilibrium theory. The examined lag time of each simulated permeation curve is demonstrated in the sixth row. As previously introduced, lag time in pure matrix is 17.8 s. The last row shows the ratio of lag time in each specimen with trapping site to the one in the specimen without trapping site. Comparing the third and the last rows, it is obvious that Equation (1) gives a reliable prediction as hydrogen occupancy in trapping site is small and Equation (2) gives better prediction as hydrogen occupancy in trapping site is higher comparing the fourth and the last rows, which indicates that both Equations (1) and (2) work well under its own prerequisites. In conclusion, generally, as the type of hydrogen trapping site is not clear, the error caused by using Equation (2) is smaller.

### 3.4. Multiple and Partial Permeation Tests

**Figure 4:**
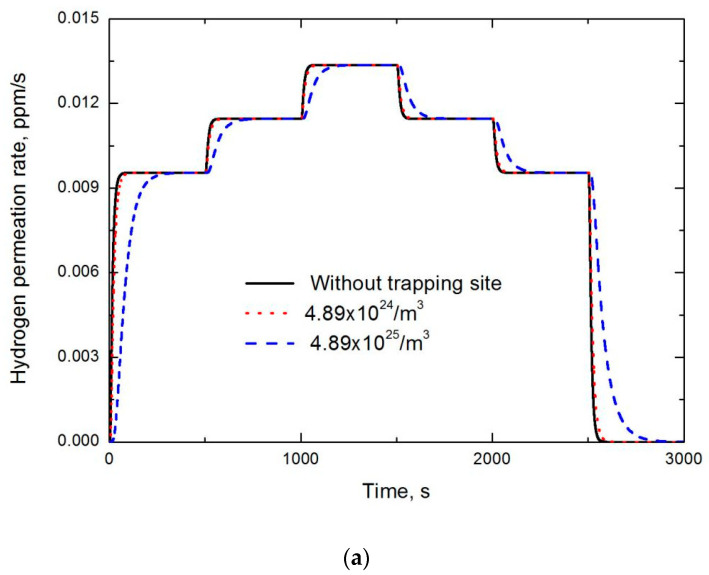

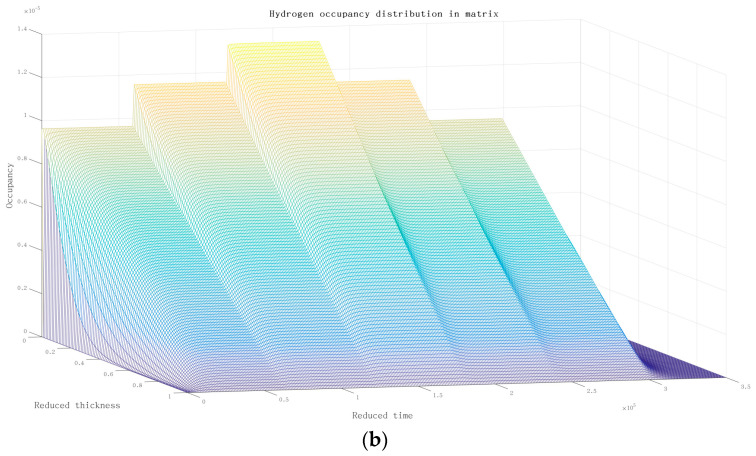
Simulated partial permeation transients varying trapping sites density and charging hydrogen concentration. (**a**) simulated permeation curves and (**b**) hydrogen occupancy distribution across the simulated specimen.

Although the above-mentioned simplified formulas theoretically work well under its own prerequisite, practical analysis on permeation curves still face problems, such as the composite effects of irreversible and reversible trapping sites, as well as specimen surface related effects. As introduced above, multiple permeation is mainly utilized to separate irreversible and reversible trapping sites. However, to further eliminate surface related conditions, partial permeation method is suggested. As shown in Figure 4a, by means of this method, hydrogen concentration at the entry side is periodically increased and then decreased. Therefore, hydrogen effective diffusivity can be examined in the second and subsequent build-up stages, instead of the first one to eliminate specimen surface effects, or in the first and subsequent decay stages instead of the last one. In Figure 4a, it is clear that the discrepancy between the permeation curves without and with considering hydrogen trapping site is increased with trapping site density. Besides its complexity, the potential problem of this method is that hydrogen occupancy in trapping sites increases with hydrogen charging concentration, as shown in Figure 4b, which could induce errors in examining trapping site density at each stage.

### 3.5. Delayed Lag Time Method

A modified Fick’s second diffusion law was also proposed to fit the experimental permeation curves [42], as shown in Equation (16).

(16) $$\text{Build-up}\;\mathrm{transient}\;\frac{i_{p}-i_{p}^{0}}{i_{p}^{\infty }-i_{p}^{0}}=\frac{2L}{\sqrt{\pi D\left(t-t_{trap}\right)}}\sum_{n=0}^{\infty }\exp\left\{-\frac{{\left(2n+1\right)}^{2}L^{2}}{4Dt\left(t-t_{trap}\right)}\right\}$$

Here, *t_trap_* is considered as the time consumed by hydrogen trapping behaviors. Obviously, Equation (16) should work in the case of irreversible hydrogen trapping sites since it demonstrates no effect on the shape of permeation curve, but retards the hydrogen permeation curve entirely, which also indicates that *t_trap_* can be further utilized to examine irreversible hydrogen trapping site density based on Equation (2). Inspired by the modified Fick’s second diffusion law, a delayed lag time method was introduced into the present work. Firstly, the traditional lag time is examined based on the entire experimental permeation curves, including the breakthrough stage. Secondly, to separate the effects of irreversible trapping sites, and eliminate specimen surface condition, a new delayed lag time could be produced by only fitting the shape characteristics of the experimental permeation curve. As shown in Figure 1, irreversible trapping sites demonstrated no influence on the shape of the permeation curve. With this delayed lag time, as well as the lag time directly examined at the entire experimental permeation curve, the irreversible trapping site’s density could be examined based on Equation (2) through the omission of specimen surface effects.

## 4. Summary

In this present work, in order to conduct hydrogen permeation test more effectively, and evaluate hydrogen trapping site characteristics more precisely, a serial of hydrogen permeation tests were numerically simulated and examined, separately considering reversible and irreversible hydrogen trapping sites. The following conclusions can be drawn:

(1) Application prerequisites of the traditional simplified methods in analyzing hydrogen trapping site characteristics should be satisfied. Equation (1) is for reversible trapping site, and Equation (2) is for irreversible trapping site.

(2) Hydrogen content examined at the decay stage only accounts for hydrogen released at the specimen exit side, without considering hydrogen released at the specimen entry side. 

(3) The method to examine irreversible trapping site density at decay stage underestimates trapping site density by about 50%. Meanwhile, a delayed lag time method is proposed to fit experimental permeation curves and analyze irreversible trapping sites density.

## Figures and Tables

**Table 1:** Input and examined parameters of simulated permeation curves considering irreversible trapping sites.

**Input Values**	Trapping sites density, 4.89 × 10^24^ m^−3^	0	1	10
	*C*_0_, part per million(ppm)	1	1	1
	1 + 3*α*/*β*	-	4.00	30.0
	1 + *α*	-	2.60 × 10^5^	2.60 × 10^6^
**Examined Values**	Lag time, s	17.8	73.2	547
	*t_T_*/*t_L_*	-	4.10	30.7

**Table 2:** Input and examined parameters of simulated permeation curves considering reversible trapping site.

**Input Values**	Trapping sites density, 4.89 × 10^24^ m^−3^	0	1	10	100	10	1	1
	*C*_0_, ppm	1	1	1	1	10	10	100
	1 + *α*	-	1.48	5.83	49.3	5.83	1.48	1.48
	1 + 3*α*/*β*	-	4.00	31.0	301	4.00	1.3	1.03
	Occupancy	-	0.33	0.33	0.33	0.83	0.83	0.98
**Examined Values**	Lag time, s	17.8	25.1	89.4	729	47.3	20.8	18.2
	*t_T_*/*t_L_*	-	1.40	5.01	41.0	2.65	1.17	1.02

## Data Availability

The raw/processed data required to reproduce these findings cannot be shared at this time as the data also forms part of an ongoing study.
